# Quality Characterization of Fava Bean-Fortified Bread Using Hyperspectral Imaging

**DOI:** 10.3390/foods13020231

**Published:** 2024-01-11

**Authors:** Sunday J. Olakanmi, Digvir S. Jayas, Jitendra Paliwal, Muhammad Mudassir Arif Chaudhry, Catherine Rui Jin Findlay

**Affiliations:** 1 Department of Biosystems Engineering, University of Manitoba, 75 Chancellors Circle, Winnipeg, MB R3T 5V6, Canada; olakanms@myumanitoba.ca (S.J.O.); mudassir.chaudhry@umanitoba.ca (M.M.A.C.); catherine.findlay@umanitoba.ca (C.R.J.F.); 2 President’s Office, University of Lethbridge, 4401 University Drive West, Lethbridge, AB T1K 3M4, Canada

**Keywords:** hyperspectral imaging, fortified bread, quality inspection, prediction, classification

## Abstract

As the demand for alternative protein sources and nutritional improvement in baked goods grows, integrating legume-based ingredients, such as fava beans, into wheat flour presents an innovative alternative. This study investigates the potential of hyperspectral imaging (HSI) to predict the protein content (short-wave infrared (SWIR) range)) of fava bean-fortified bread and classify them based on their color characteristics (visible–near-infrared (Vis-NIR) range). Different multivariate analysis tools, such as principal component analysis (PCA), partial least square discriminant analysis (PLS-DA), and partial least square regression (PLSR), were utilized to assess the protein distribution and color quality parameters of bread samples. The result of the PLS-DA in the SWIR range yielded a classification accuracy of ˃99%, successfully classifying the samples based on their protein contents (low protein and high protein). The PLSR model showed an RMSEC of 0.086% and an RMSECV of 0.094%. Also, the external validation resulted in an RMSEP of 0.064%. The PLSR model possessed the capability to efficiently predict the protein content of the bread samples. The results suggest that HSI can be successfully used to classify bread samples based on their protein content and for the prediction of protein composition. Hyperspectral imaging can therefore be reliably implemented for the quality monitoring of baked goods in commercial bakeries.

## 1. Introduction

Bread is one of the most commonly consumed foods globally. The availability of diverse ingredients and recipes accounts for their consumption in different shapes and forms by different people [1,2]. In addition to water, sodium chloride, and leavening agents, wheat flour is most commonly used for bread production, owing to wheat’s excellent viscoelastic properties. In the past few years, consumers have grown increasingly aware of product quality, which has increased the desire for healthy bread types, especially protein-rich, fiber-rich, and gluten-free breads. Because of the nutritional deficiencies of wheat flour, particularly refined/white flour, which is often used for bread production, considerable efforts have been made by the research community to produce bread types that are rich in protein, fiber, and other health-promoting bioactive compounds like vitamins, flavonoids, polyphenols, and β-carotene. Pulse flours are a rich source of protein and fiber, with the ability to complement the essential amino acids present in wheat flour, especially sulphur-containing amino acids. These flour blends have been used to produce wheat flour-based baked goods [1,3,4,5,6]. Fava beans (*Vicia faba* L.) are a grain legume with great agronomic and nutritional importance. Depending on the variety, fava bean consists of 26–33% protein (with leucine and lysine amino acids in the ranges of 50.8–72.1 and 44.8–74.8 mg/g protein, respectively), soluble fiber in the range of 0.55–1.06%, insoluble fiber in the range of 10.7–16.0%, iron in the range of 1.8–21.3 mg/100 g, and zinc in the range of 0.9–5.2 mg/100 g. Compared to other legumes, fava beans contain higher amounts of phosphorous, calcium, zinc, and iron [7,8,9]. However, despite its nutritional importance, its utilization for human food has been limited.

Important factors that determine a consumer’s purchase of bread include the attractiveness of its color and nutritional profiles. As with other food products, the information on the nutritional value of bread labels is required to be accurate and reliable to meet the regulatory requirements [10,11,12,13]. The protein content in a bread sample is one of the determinants of its nutritional quality. The protein content is often expressed as its amount per 100 g of the whole loaf, with the crust, and as a function of the dry matter [10]. Conventionally, the color is assessed using a colorimeter. The protein content is estimated using the Kjeldahl and Dumas combustion methods, which often involve lengthy and multi-stage processes. These techniques are often destructive, laborious, error-prone, and require considerable user input and time [10,14,15]. Also, these techniques are unsuitable for the real-time assessment of these quality parameters, which is often desirable in present-day industries. Therefore, there is a need for green and non-destructive techniques that can provide a fast and accurate assessment of the quality parameters.

Hyperspectral imaging (HSI) is one of the most widely used, non-destructive techniques to assess the quality attributes of food commodities. It combines the principles of spectroscopy and image processing to provide spectral and spatial information about a sample. This technique has proven economical, fast, reliable, and less laborious for the quality evaluation of food products, providing details about their states, concentrations, and compositions [16,17,18,19,20]. In combination with different multivariate analysis tools, e.g., partial least square regression (PLS-R), principal component analysis (PCA), the partial least squares discriminant analysis (PLS-DA) method, linear discriminant analysis (LDA), and support vector machine (SVM), HSI has been used to quantify different macromolecules in different baked products. Examples include the moisture contents of baguette slices after 96 h of storage (950–2495 nm) [21], bread [22], and biscuits (950–2500 nm) [23,24]; fats and moisture content of doughnuts (1100–2100 nm) [25]; the moisture content and hardness of cakes (900–1700 nm) [26]; and the water activity prediction of mamón cakes (Filipino sponge) (935–1720 nm) [27]. Other studies have used HSI to monitor the safety and shelf-life of different baked goods, for example, mold detection in bread slices after 7 and 21 days of storage [28,29] and in cake (935–1720 nm) [30,31]. HSI has also been utilized to achieve various objectives, such as pulse flour classification based on protein and color (400–1000 nm and 1000–2500 nm) [15], segregation of bison muscles based on color stability [32], classification of pork loin based on brine concentration (450–1664 nm) [33], identification of different particle sizes of milk powders [34] (400–1000 nm), discrimination of maize kernels (874–1733 nm) [18], and hardness prediction of wheat kernels [35].

Considering the successful application of HSI for assessing the quality and safety of different agri-food products, the objective of the present study is to use the HSI technique to characterize and classify fava bean-fortified bread samples based on the color and protein content. The research investigates the effects of particle sizes of fava bean flour and the substitution level to wheat flour on bread’s protein and color attributes. We acquired the images in the Vis-NIR (400–1000 nm) and SWIR (1000–2500 nm) regions to classify the bread samples based on the color and protein quality attributes. Furthermore, wavelength selection was performed to identify the most relevant variables capable of classifying bread based on low- and high-protein contents and to predict the protein content in bread. This research aims to assist food processors in efficiently and rapidly predicting protein content in bread for labeling purposes and to assist food inspection agencies.

## 2. Materials and Methods

### 2.1. Materials

#### 2.1.1. Flour Preparation

A whole grain of low-vicine/convicine fava bean (*Vicia faba* L.) of the variety Fabelle used for this study was sourced from Prairie Fava, Winnipeg, Canada. The grain was harvested in 2022 and dehulled using an impact dehuller (DOSB model, Bühler Group, Uzwil, Switzerland). Before using the desired screen sizes for the milling process, a prebreak process was carried out on the dehulled/split seeds using a Jacobson hammer mill (model 120-B; Jacobson Machine Works Inc., Davenport, IA, USA) fitted with a 3.00 mm screen size. The prebreak seeds were milled using a single-stage Ferkar multipurpose knife mill (Ferkar model 5; KFM, d.o.o., Velenje, Slovenia) fitted with 0.14, 0.50, and 1.0 mm screen sizes, representing fine, medium, and coarse flours, respectively, set at 50 Hz motor, with a feed rate of 8 kg/h. The seeds were not tempered before the milling process. The dehulling and milling processes were conducted at Cereals Canada in Winnipeg, Canada. The wheat (*Triticum aestivum*) flour blended with the fava bean flour was milled by utilizing a Bühler MLU 202 lab roller mill (Bühler Group, Uzwil, Switzerland). The flour was derived from Canada Western Red Spring (CWRS) wheat graded No. 1 and grown in 2022. The milled flours were kept in polyethylene bags and stored in a temperature (15 °C)- and humidity (60%)-controlled environment for the bread production process.

#### 2.1.2. Bread Production

For the bread production, the fava bean flour at the three particle sizes (i.e., 0.14, 0.50, and 1.00 mm) was blended with wheat flour at 10, 20, and 30% substitution levels (referred to as SL10, SL20, and SL30). The no-time dough method (NTD) [36] was used for the test baking and was carried out in the pilot bakery at Cereals Canada. Table 1 shows the mixing formula for the pan bread. The flour blends are a mix of wheat and fava bean flours. All other ingredients were added as a percentage of the weight of the flour blends.

**Figure 1:**
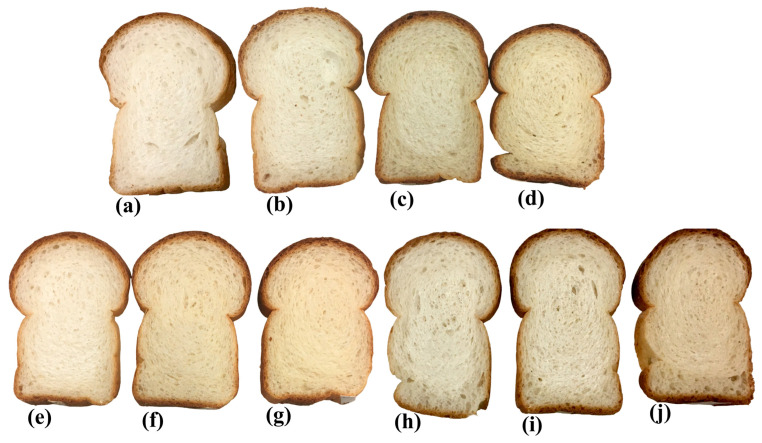
Bread samples: (**a**) control, (**b**) 0.14 mm SL10, (**c**) 0.14 mm SL20, (**d**) 0.14 mm SL30, (**e**) 0.5 mm SL10, (**f**) 0.5 mm SL20, (**g**) 0.5 mm SL30, (**h**) 1.0 mm SL10, (**i**) 1.0 mm SL20, and (**j**) 1.0 mm SL30.

All ingredients were placed in a 100–200 g spiral mixer (National Manufacturing Co., Inc., Piscataway, NJ, USA) and mixed at a slow speed for 2 min and then at a fast speed until optimum gluten development (5–8 min; up to about 10% after the peak). As shown in Table 1, 4% gluten was added to all the flour mixes except for the SL10 for the three particle sizes. The temperature of the dough was recorded using a laboratory-scale thermometer. The dough was allowed to rest for 10 min and then scaled into two pieces of 165 g per piece. The dough was kneaded into a ball by hand, placed into a lightly greased fermentation bowl, covered, and rested for another 10 min at an ambient temperature. The dough ball was sheeted three times in succession at 5/16, 3/16, and 1/8 inches using a sheeter (National Manufacturing Co., Inc., Piscataway, NJ, USA). The sheeted dough was placed on a plastic bench of the molder and rolled. The molder (National Manufacturing Co., Inc., Piscataway, NJ, USA) was set at a 130 mm lane and a 130 mm roller gap, with 62 mm bent pins. The top roll was pulled down and molded for 10 s. The dough was then placed into baking pans (approximately 550 mL in volume) with the following dimensions: top (inside) 14.5 cm × 8.0 cm, bottom (outside) 12.8 cm × 6.4 cm, and depth 5.7 cm. The baking pan was placed into a fermentation cabinet (National Manufacturing Co., Inc., Piscataway, NJ, USA) set at 85% relative humidity and a 37 °C temperature to a height of 90 mm (using a proof height gauge; Mitutoyo Absolute Digimatic Height Gauge; Mitutoyo America Corp. Aurora, IL, USA), and the proofing time was recorded. The baking pan was carefully placed into an electric reel oven (National Manufacturing Co., Inc., Piscataway, NJ, USA) and baked at 204 °C for 20 min. A 100% wheat flour sample was baked daily, serving as the control (denoted as CTRL). The bread samples were sliced (Figure 1) and stored in a freezer (−25 °C) for further analyses.

### 2.2. Methods

#### 2.2.1. HSI Imaging System, Image Acquisition, and Correction

**Figure 2:**
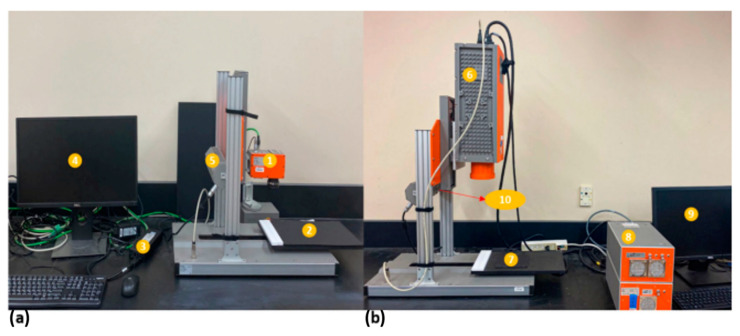
(**a**) Integrated Vis-NIR HSI system: 1—spectrograph, 2—moving stage, 3—control panel, 4—computer system, and 5—lighting system (halogen lamps). (**b**) Integrated SWIR HSI system: 6—spectrograph, 7—moving stage, 8—control panel, 9—computer system, and 10—lighting system (halogen lamps).

The frozen bread slices were taken out of the freezer, cut into six equal sizes using a sharp knife, and allowed to thaw at atmospheric conditions before HSI acquisition. The bench-top line-scanning HSI systems (SPECIM Spectral Imaging Ltd., Oulu, Finland) were utilized to capture the images. Figure 2 shows the arrangement of the two systems comprising the Vis-NIR (400–1000) and SWIR (1000–2500) HSI systems. The Vis-NIR system consisted of a V10E spectrograph (SPECIM, 397.66–1003.81 nm with a spectral resolution of 2.6 nm), a charge-coupled device (CCD) camera with a 1024 × 896 pixel spatial resolution, and a focusing lens (OLET 15). The system also contained two 150 W 3900-ER tungsten lamps (Illumination Technology Inc., Liverpool, NY, USA) at 45° to the sample holding stage in the illumination section (Figure 2a). For the SWIR system, the components included a camera and a mercury–cadmium–tellurium detector joined with an N25E spectrograph (SPECIM Spectral Imaging Ltd., Oulu, Finland) and a focusing lens (OLES30; SPECIM Spectral Imaging Ltd., Oulu, Finland) with a spatial resolution of 300 × 384 × 288 pixels. Similar to the Vis-NIR system, the imaging unit contained a moving stage with a sample tray, a desktop computer (Tower 3620, Dell, Round Rock, TX, USA) with the Lumo software Suite (SPECIM Spectral Imaging Ltd, Oulu, Finland) installed for acquiring images, motor control, and a lighting setup (Figure 2b) [32,37].

In order to achieve temporal and thermal equilibrium of the camera and the lighting system, the systems were turned on for 30 min before scanning the samples [15,35]. The frame rate for the two cameras was set at 20 frames/s. Also, the speed of the moving stage of the two systems was set at 7 mm/s to achieve the best aspect ratio of the exposure time and the frame rate [35]. For the SWIR HSI system, we adjusted the exposure time to 8 ms, while it was set at 20 ms for the Vis-NIR system. To obtain a black reference, the shutter was closed automatically. Also, we positioned a 99% Spectralon reflectance standard (Labsphere, North Sutton, NH, USA) at the upper part of the white reference for every image. After acquiring every image, the white and black reference corrections were applied.

#### 2.2.2. Image Processing and Spectral Extraction

The HSIs in the SWIR and Vis-NIR ranges were segmented using the Otsu technique [38]. During the segmentation process, we developed binary images, with the pixels of the unwanted background set at zero (black), and the nonzero (white) elements were utilized to extract the pixels of the bread samples. The average of the spectra of the pixels of the bread samples for each image was obtained to acquire a representative spectrum. For the Vis-NIR region, images at 526 nm were utilized to segregate the pixels of concern from the background. For the SWIR region, the best contrast between the pixels of the bread sample and their background was obtained at 1513 nm. Also, the wavebands with the smallest amount of signal-to-noise ratio were removed. The binary images were applied to all the 224 and 288 waveband images for the Vis-NIR and SWIR wavelength regions, respectively. The spectral data obtained from each bread sample was arranged in an Excel file for multivariate data analysis. MATLAB software (MATLAB version 9.6, R2019a, MathWorks Inc., Natick, MA, USA) was used for the image segmentation and spectral data extraction [32].

#### 2.2.3. Multivariate Data Analysis

The multivariate data analysis used the PLS toolbox software (version 8.7.1) compatible with MATLAB (MATLAB version 9.6, R2019a, MathWorks Inc., Natick, MA, USA). The study aimed to utilize the spectral characteristics of the bread samples produced from wheat flour substituted with fava bean flour at three particle sizes (0.14, 0.5, and 1.0 mm) and three substitution levels (SL10, SL20, and SL30) to explore the feasibility of classification based on these parameters and for protein prediction. Before applying the multivariate data analysis techniques, different mathematical pretreatments, such as first and second derivatives, mean centering, standard normal variate, and smoothing, were employed to preprocess the spectral datasets.

The multivariate analysis tools employed for outlier removal, dimensionality reduction, and model development in this study were principal component analysis (PCA) and partial least squares regression (PLS-DA). PCA is an algorithm applied for the dimensionality reduction of the data into a set of statistically uncorrelated orthogonal variables called principal components (PC). The PCA model was utilized to describe the unevenness in the datasets acquired from the bread samples. PLS-DA is a supervised technique employed for classification to obtain the optimum discrimination between distinct sample groups. It models the data into a new variable set called latent variables (LVs), with the first few LVs explaining the maximum co-variance between the response variables, including the dummy variables and a design matrix depicting the different groups in the dataset [15,18,32,34].

Supervised PLS-DA classification was performed with two classes: high protein and low protein. The bread samples from CTRL and SL10 were considered low protein, whereas SL20 and SL30 were categorized as high protein. Before applying PLS-DA, the spectral datasets in the SWIR regions were grouped into calibration and external validation/prediction sets utilizing a supervised tool called the Kennard–Stone algorithm, with 70% of the data for calibration and 30% for external validation/prediction. The PLS-DA model’s performance was evaluated using the confusion matrices, specificity (SPEC), and sensitivity (SENS) values. The model sensitivity was expressed as the proportion of the true positives to the addition of total false negatives and true positives. The model specificity was defined as the ratio of the true negatives to the addition of total false positives and true negatives. The model accuracy was expressed as the proportion of accurately categorized samples (negatives or positives) to the total number of samples in the dataset [32].

For the SWIR datasets, 56 samples were used to develop calibration models, and contiguous block cross-validation was utilized with 6 data splits. The set of 24 samples used for the prediction/external validation was used to evaluate the calibration model in an external prediction. Different partial least squares regression (PLSR) models were developed to select the best model for predicting protein, and their performance was compared. Initially, a model was developed using all 288 wavelengths in the SWIR range. Interval-based PLS (iPLS) was utilized by setting particular intervals (intervals = 3; interval width = 5) in the SWIR region to select a wavelength adequate for protein prediction. Variable importance in projection (VIP) scores was another approach used for the wavelength selection. After developing the models, their performance was assessed, the best-performing calibration model was chosen, and external validation was performed. The R^2^ value was used to select the best calibration model, with the best model having the highest R^2^ value in the calibration, cross-validation, and external validation and the lowest root mean square errors (RMSE) in calibration (RMSEC) and cross-validation (RMSECV) [32].

#### 2.2.4. Protein Content and Color Determination

The objective assessment of the crumb color of the bread samples was carried out using a Minolta (Chroma Meter CR-410, Minolta Co., Ltd, Tokyo, Japan) and calibrated prior to use by scanning the standard white tile. The CIE L* (means lightness), a* (means redness), and b* (means yellowness) values of each sample were determined. The protein content (%) (*N* × 6.25, where *N* represents the nitrogen content) of the bread samples was estimated using the LECO FP-628 (LECO Corporation, St Joseph, MI, USA). Daily drift corrections were performed using ethylenediaminetetraacetic acid (EDTA) [39]. Prior to the protein analysis, the samples were dried using a laboratory oven set at 103 °C for 4 h, allowed to cool in a desiccator, and ground into powder. The results from the objective measurements were analyzed using the SPSS software (IBM statistical analysis version 25.0), and the significance among the samples was compared at *p* ≤ 0.05 using Duncan’s post-hoc comparison test. Thirty-six tests were carried out for each of the samples, and the average was recorded. The results of the objective measurements of the protein and color measurements were correlated with the spectral profiles obtained from the hyperspectral imaging technique.

## 3. Results and Discussion

### 3.1. Analytical Techniques (Color and Protein Determination)

**Figure 3:**
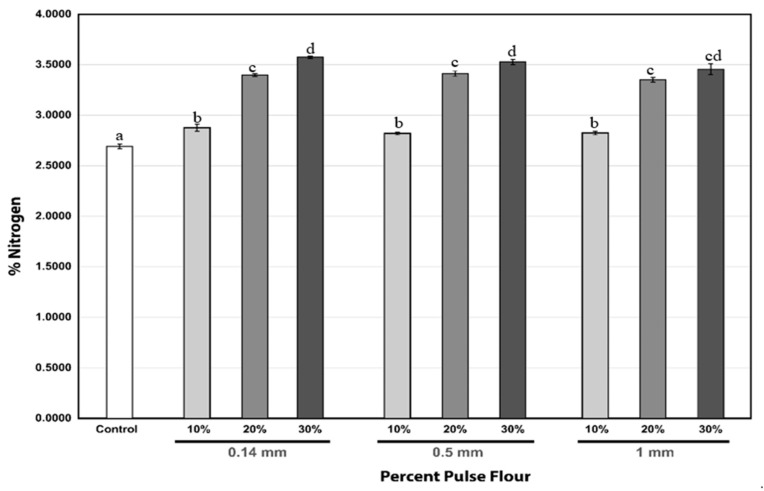
Variations in the nitrogen content (%) of the bread with different particle sizes and substitution levels. Values marked with different letters are significantly different (*p* ≤ 0.05). Error bars represent ± standard error (n = 36).

The nitrogen content (%) of the different flour combinations and particle sizes are depicted in Figure 3. The CTRL samples possessed the lowest nitrogen content (%), followed by the SL10, across all three particle sizes. It can also be observed that nitrogen content (%) increased statistically significantly, transitioning from SL10 to SL30 across all particle sizes. The highest increase in the nitrogen content (0.55%), from an average of 2.85 ± 0.03 to 3.39 ± 0.03% after considering the pooled results from the three particle size types, occurred when the percentage of pulse flour was increased from SL10 to SL20 in the bread. However, there were diminishing returns regarding nitrogen content (%), as a further increase in pulse flour percentage from SL20 to SL30 resulted in a lower increase of only 0.13% more nitrogen, from an average value of 3.39 ± 0.03 to 3.52 ± 0.06%. From the figure, it can also be deduced that varying the particle sizes of the fava bean flour did not have a significant impact on the nitrogen content (%) of the bread.

### 3.2. Spectral Profiles in the SWIR and Vis-NIR Ranges of the Dataset

**Figure 4:**
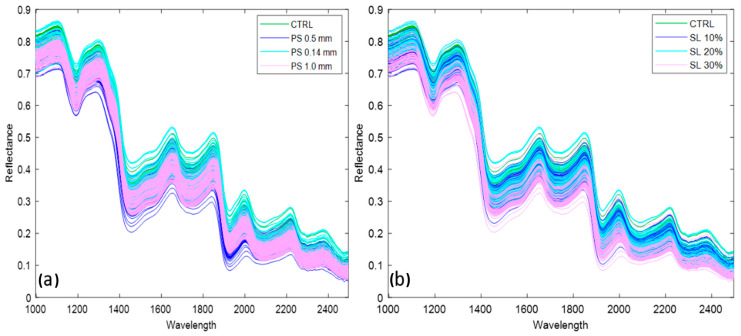
Spectral profiles of the bread samples: (**a**) colored by particle size; (**b**) colored by substitution levels.

Figure 4a,b shows the raw spectra acquired from the bread dataset grouped by particle size and substitution level for the SWIR range, respectively. Peaks in the spectral profiles at 1250–1350 nm, 1400–1500 nm, 1600–1800 nm, and 1900–2100 nm can be associated with different macromolecules in the bread samples. The C-H structure at 1329 nm denotes the presence of carbohydrates, 1400 nm depicts the O-H structure in the first overtone, and 1450 nm and 1960 nm indicate the presence of moisture. Moreover, the RNH_2_ stretching at 1530 nm denotes the presence of protein content in the bread samples. For the Vis-NIR regions, some peaks were observed in the 650–750 nm wavelength related to the presence of the red color [15,32,40,41,42].

### 3.3. Principal Component Analysis (PCA)

**Figure 5:**
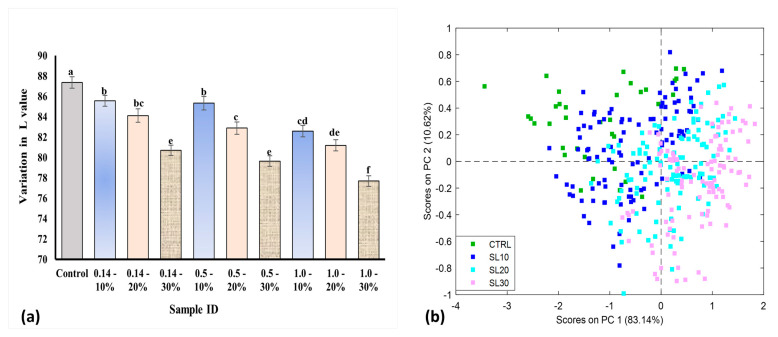
(**a**) Lightness color values; (**b**) PCA scores plot grouping the samples based on color. Values marked with different letters are significantly different (*p* ≤ 0.05). Error bars represent ± standard error (n = 36).

PCA models were developed for both Vis-NIR and SWIR datasets. In the case of the Vis-NIR, the PCA model yielded a slight trend in groupings based on the color changes in the bread samples possessing different flour compositions. The samples were distinguished along the PC1 axis (covering 83.14% of the variance in the data), as shown in Figure 5a. CTRL and SL10 had similar color characteristics, whereas SL20 and SL30 were grouped. This can also be confirmed by the lightness values (L*) from the Lab color space, as shown in Figure 5b. Studies have shown that the regions between 450 nm and 670 nm are mainly responsible for chromophore development in different food products [15,43,44]. It could be observed that for each of the particle sizes, the L* values decreased statistically significantly with increasing substitution levels. This is consistent with the studies reported in literature that show that adding pulse flour to wheat flour results in bread with a darker color associated with decreasing L* values. This color change occurs due to the lysine in the pulse flour. Lysine is an essential amino acid that reacts with reduced sugar to produce brownish coloration during the Maillard reaction [6,45,46,47,48].

**Figure 6:**
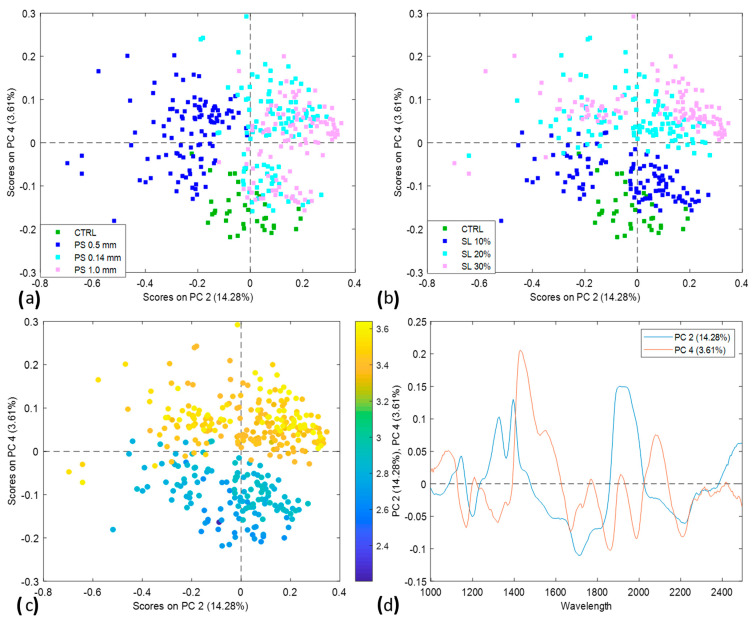
(**a**) PCA scores plot colored by particle sizes; (**b**) PCA scores plot colored by substitution levels; (**c**) PCA scores plot colored by protein content; (**d**) loadings plot for PC2 and PC4.

In the SWIR wavelength range, the PCA model yielded thorough insights, grouping bread samples based on substitution levels and particle sizes, as shown in Figure 6a,b. Figure 6c,d depicts the PCA scores plot colored by protein and the loading plots of PC2 vs. PC4, respectively.

It can be deduced from Figure 6a that the bread samples with a 0.5 mm particle size were discriminated from the control and other particle sizes. This can be attributed to the relationship between diffuse reflectance and the porosity of the bread samples, indicating a higher porosity in the 0.5 mm particle size bread samples than in the other two combinations [49,50,51,52]. However, when the samples were grouped based on the substitution levels, PC4 grouped CTRL and SL10 classes as one group and SL20 and SL30 as a separate group. This implies that increasing the fava bean substitution level led to a corresponding increase in the protein content of the bread. Comparing the results from Figure 3 and Figure 6c, it can be observed that CTRL and SL10 would be grouped as one group, whereas SL20 and SL30 would be grouped as separate groups corresponding to the differences in their respective protein contents.

### 3.4. Supervised Classification Using Partial Least Squares Discriminant Analysis (PLS-DA)

**Figure 7:**
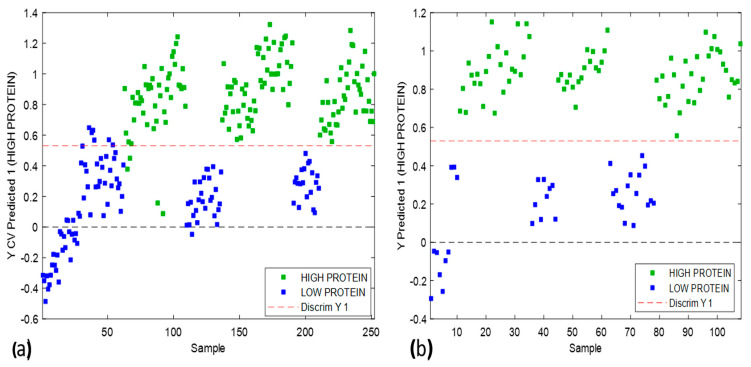
(**a**) PLS-DA calibration model scores plot; (**b**) PLS-DA external validation model scores plot.

The model that depicted the best results was pretreated with mean centering only with classification accuracies of >98% in calibration and cross-validation, followed by an external prediction accuracy of 99%, as shown in Figure 7a,b.

For the calibration model, the samples from low- and high-protein classes were 100% correctly classified in their respective classes using 15 out of 288 variables in the SWIR range using the i-PLS approach. The wavebands used to develop this PLS-DA classification model were associated with the N-H overtones in the SWIR wavelength range, which can be related to the LECO measurements, which measure the nitrogen content before calculating protein content. In the case of cross-validation, the model showed a 99% classification accuracy for the low-protein class and 100% accuracy for the high-protein class. The results were similar for external validation, as shown in the confusion matrix in Table 2.

It can be observed from Table 2 that the values of class sensitivity and specificity for calibration, cross-validation, and external prediction were considerably high, which is an indication of accurate classification. Similar results were reported by Sivakumar et al. (2022), where the authors used PLS-DA to classify pulse flours based on milling methods and pulse types [15]. The authors identified some wavebands that were associated with the fat and moisture attributes of the flours and possessed significant weightage in the model. Hence, it can be concluded that PLS-DA with wavelength selection can serve as an effective tool for classifying bread samples based on protein content.

### 3.5. Protein Content Prediction Using Partial Least Squares Regression (PLSR)

**Figure 8:**
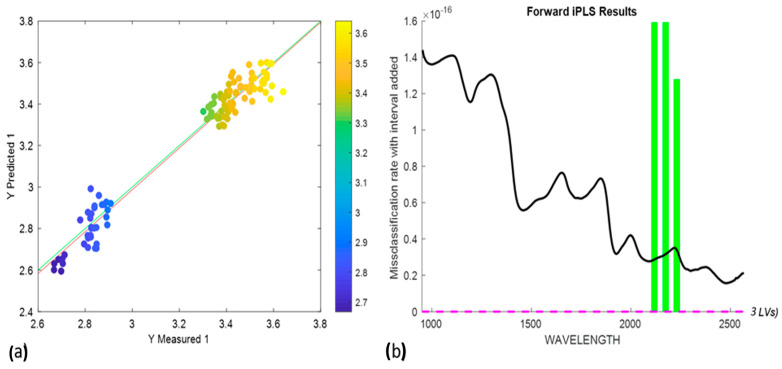
(**a**) PLS regression plot for protein content prediction; (**b**) i-PLS-based variable selection plot. The black line represents the whole spectra used while the green lines represent the selected wavelength for the prediction.

The protein content prediction model was developed using the PLSR. Out of a total of 288 variables, 15 variables were used to develop the prediction model. The PLSR model was developed using the SIMPLS algorithm with mean centering as preprocessing, resulting in 4 LVs. The variables were selected using forward interval-based PLS (i-PLS), as shown in Figure 8b. The model resulted in an $R_{cal}^{2}$ of 0.94, $R_{cv}^{2}$ of 0.92, RMSEC of 0.086%, and RMSECV of 0.094%. The external validation resulted in an $R_{pred}^{2}$ of 0.959265 and an RMSEP of 0.064%. Figure 8a,b represents the PLS regression and i-PLS interval plots, respectively.

This study confirms the prediction capability of the PLSR model to quantify different macromolecules in food products, for example, protein composition and their secondary structures [53,54,55,56,57,58,59,60]. Wilcox et al. (2016) reported that the PLSR model was able to predict the secondary structure of protein FTIR spectra data in the Amide 1 and II regions with RMSEPs of about 12%, 7%, 7%, and 8% for α-helix, β-sheet, antiparallel β-sheet, and other conformations, respectively [61]. Mahesh et al. (2015) reported that PLSR outperformed the principal components regression (PCR) model for the prediction of protein contents and hardness values of wheat, with RMSEP, RSECV, and r values of 1.76, 1.33, and 0.68, respectively, as compared with PCR, which presented 147.7, 12.15, and 0.82 for the same variables [62]. In another study, the PLSR model showed a lesser accuracy, when compared with Bayesian methods, at predicting the fatty acid and protein compositions of different dairy products. However, the accuracy was negligible, with the differences in the predictions between the models being less than 1% accuracy [63].

## 4. Conclusions

This study employed near infrared HSI to predict and classify bread samples based on their protein content and color characteristics. This information can be utilized by the food industry for labeling purposes to accommodate legislative requirements. The food inspection agencies can also possibly use this information for the quality monitoring of value-added bread. The outcomes from the PLS-DA in the SWIR range exhibited significant precision for the classification of bread samples based on their protein contents. Out of a total of 288 wavelengths, only 15 were selected for the development of the PLS-DA and PSLR models. The wavebands from 2107–2129 nm, 2163–2185 nm, and 2219–2241 nm can be associated with the nitrogen content, hence representing protein. Additionally, the PLSR model provided robust results in the same waveband regions for the prediction of protein content. This study explicitly establishes HSI as a promising and effective technique for the real-time assessment of baked goods, emerging as a valuable tool for quality control processes. Some of the limitations of the HSI technique include the expertise required to periodically calibrate the system and the influence of ambient lighting. Moreover, these models can only be used for the specific samples under consideration, as the variance captured by the models is limited. In order to enhance the prediction and classification capabilities of the model, they need to be updated to capture a wider variance. Looking forward, avenues for future research could explore advanced deep learning techniques to further enhance the reliability and accuracy of diverse models within this domain. Also, these models should be applied to different products with varying physical and chemical properties. This will further enhance the accuracy and suitability of HSI in food quality control and inspections.

## Figures and Tables

**Table 1:** Dough mixing formula for the pan bread.

Ingredients	Mixing Proportions (%)
Flour blends (14%, db)	100
Water	Farinograph + 1%
Yeast, fresh	4.0
Sugar, refined	4.0
Salt, refined	1.2
Whey, powder	4.0
Shortening	3.0
Ammonium phosphate	0.1
Doh-Tone 2	0.03
Ascorbic acid	60 ppm
Gluten	4.0

**Table 2:** PLS-DA statistics for the classification of bread samples based on protein content.

**Calibration**		**Actual Class**		**N**	**Global**	
		**High Protein**	**Low Protein**		SENS	SPEC
**Predicted class**	**High protein**	142	0	142	1.000	1.000
	**Low protein**	0	109	109	1.000	1.000
	**Total**			251		
					
**Cross Validation**		**Actual class**		**N**	**Global**	
		**High protein**	**Low protein**		SENS	SPEC
**Predicted class**	**High protein**	142	1	142	1.000	0.991
	**Low protein**	0	108	109	0.992	1.000
	**Total**			251		
					
**External Prediction**		**Actual class**		**N**	**Global**	
		**High protein**	**Low protein**		SENS	SPEC
**Predicted class**	**High protein**	72	0	73	0.986	1.000
	**Low protein**	1	35	35	1.000	0.986
	Total			108		

## Data Availability

All relevant data used in this manuscript can be accessed through the corresponding authors.
